# Gynura segetum-induced liver injury leading to acute liver failure: a case report and literature review

**DOI:** 10.1186/s12906-022-03549-6

**Published:** 2022-03-08

**Authors:** Zilong Zhang, Haibo Zou, Zonglin Dai, Jin Shang, Shining Sure, Chunyou Lai, Ying Shi, Qinyan Yang, Guangming Xiang, Yutong Yao, Tianhang Feng, Deyuan Zhong, Xiaolun Huang

**Affiliations:** 1 Department of Hepatobiliary-Pancreatic Surgery, Cell Transplantation Center, Sichuan Provincial People’s Hospital, University of Electronic Science and Technology of China, Chengdu, China; 2 Chinese Academy of Sciences Sichuan Translational Medicine Research Hospital, Chengdu, China; 3 Department of Pathology, Sichuan Provincial People’s Hospital, University of Electronic Science and Technology of China, Chengdu, China

**Keywords:** Gynura segetum, Drug-induced liver injury, Acute liver failure, Case report

## Abstract

**Background:**

Gynura segetum (GS) is widely used in medical care and in community settings in China as the herbal remedy. It is widely thought to have antiphlogistic properties and pain relief in traditional Chinese medicine. It has been reported that GS can cause chronic drug-induced liver injury (DILI), manifested as hepatic sinusoid obstruction syndrome (HOSO). But case reports of acute DILI developing acute liver failure (ALF) due to GS are extremely rare.

**Case presentation:**

We report a case of a 63-year-old female patient with hepatolithiasis for more than 6 years. There were no deterioration of liver function and no history of viral liver disease, autoimmune liver disease, blood transfusion or surgical allergy before operation. ALF and grade II liver encephalopathy occurred after partial hepatectomy. To follow up the medical history, the patient has been taking GS (Tusanqi) for a year and a half. The causality assessment was done by the updated Roussel Uclaf Causality Assessment Method, and the possibility of DILI caused by GS as highly probable for the score was 6 points. Excluding other causes, a diagnosis of DILI-associated ALF was established. After symptomatic support and artificial liver support system (ALSS) treatment, the clinical symptoms and signs of the patients were significantly improved. After discharge, the liver function of the patients returned to normal.

**Conclusions:**

Based on this rare case of severe liver injury, we recommend that timely prevention, identification, and appropriate management of DILI is essential for patients with a history of taking GS and other hepatotoxic drugs, and careful monitoring of liver function for patients with DILI could avoid ALF as far as possible.

## Background

Gynura segetum (Tusanqi or Jusanqi) is an erroneous species substitute of the Traditional Chinese medicines herb Sedum aizoon, and it has been widely used in Chinese folk medicine and plays a role in promoting microcirculation and relieving pain. However, its hepatic toxicity is often overlooked, and it has been reported that GS could cause DILI [1–3].

GS-induced DILI involves fibrous obliteration and destruction of the central veins and sub-lobular venules, leading to cirrhosis [3]. The clinical characteristics of GS-induced DILI are not typical, its diagnosis is very difficult and is mainly based on a careful history and exclusion of other causes. A few cases of DILI relating to GS usage have been reported in literature. However, GS-induced DILI developing into ALF has not been reported. The diagnostic strategy, management, and prognosis of GS-induced DILI are rarely described.

In this case report, we report a case of a patient with severe GS-induced DILI who developed ALF and was successfully resuscitated by ALSS and reviewed the papers related to GS-induced DILI in the literature.

## Case presentation

**Fig. 1:**
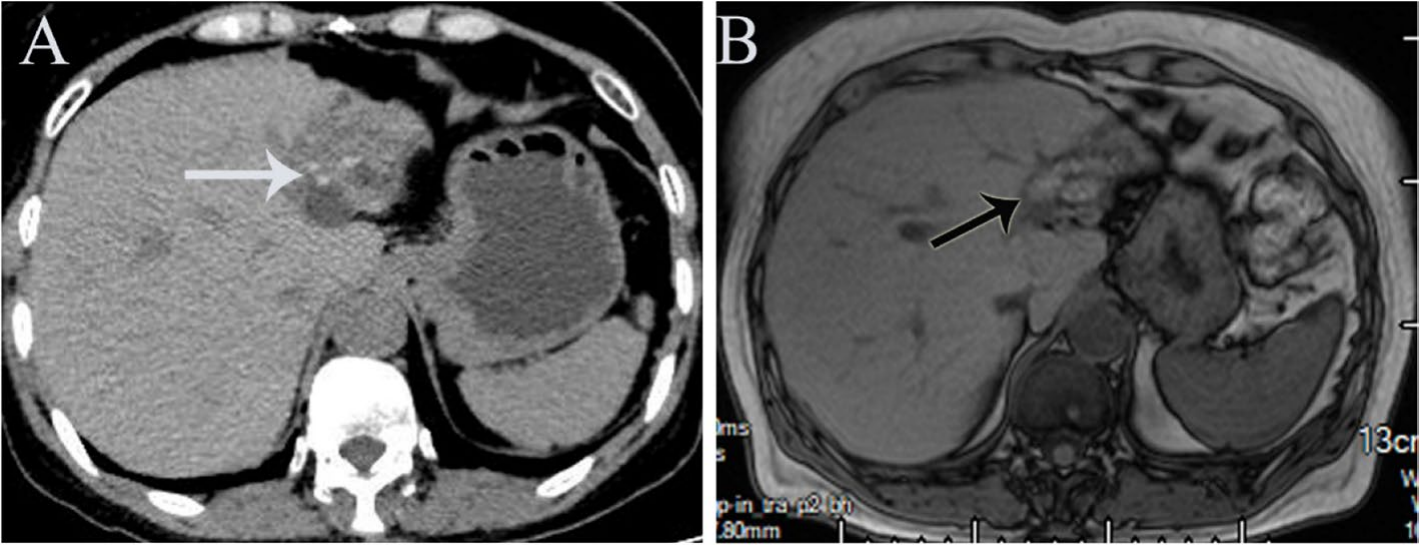
Preoperative imaging workup of left intrahepatic bile duct calculi and hepatic atrophy A the upper abdominal enhanced CT showing hepatolithiasis with dilatation of right-sided intrahepatic bile ducts (white arrow). B Contrast-enhanced MRI further delineated the anatomy of atrophy of the left lateral lobe of the liver (black arrow)

A 63-year-old woman with a history of hepatolithiasis for more than six years was suffering from intermittent low back pain. She was followed up in a local hospital for a long time, though the treatment was not effective. The patient was reexamined in the local hospital 20 days ago. The upper abdominal enhanced CT revealed intrahepatic bile duct stones with extrahepatic bile duct dilatation, atrophy of the left lobe of the liver, cholestasis of the left lobe of the intrahepatic bile duct and no sign of portal hypertension (Fig. 1A). She was admitted to Sichuan Provincial People’s Hospital for further diagnosis and treatment. Clinical history documented hypertension in the patient. The patient also denied drug use, trauma, surgery, alcohol abuse, and blood transfusion and claimed no history of other systemic diseases, no recent travel history, no mushroom ingestion, no family history of similar hepatolithiasis or any liver disease.

**Fig. 2:**
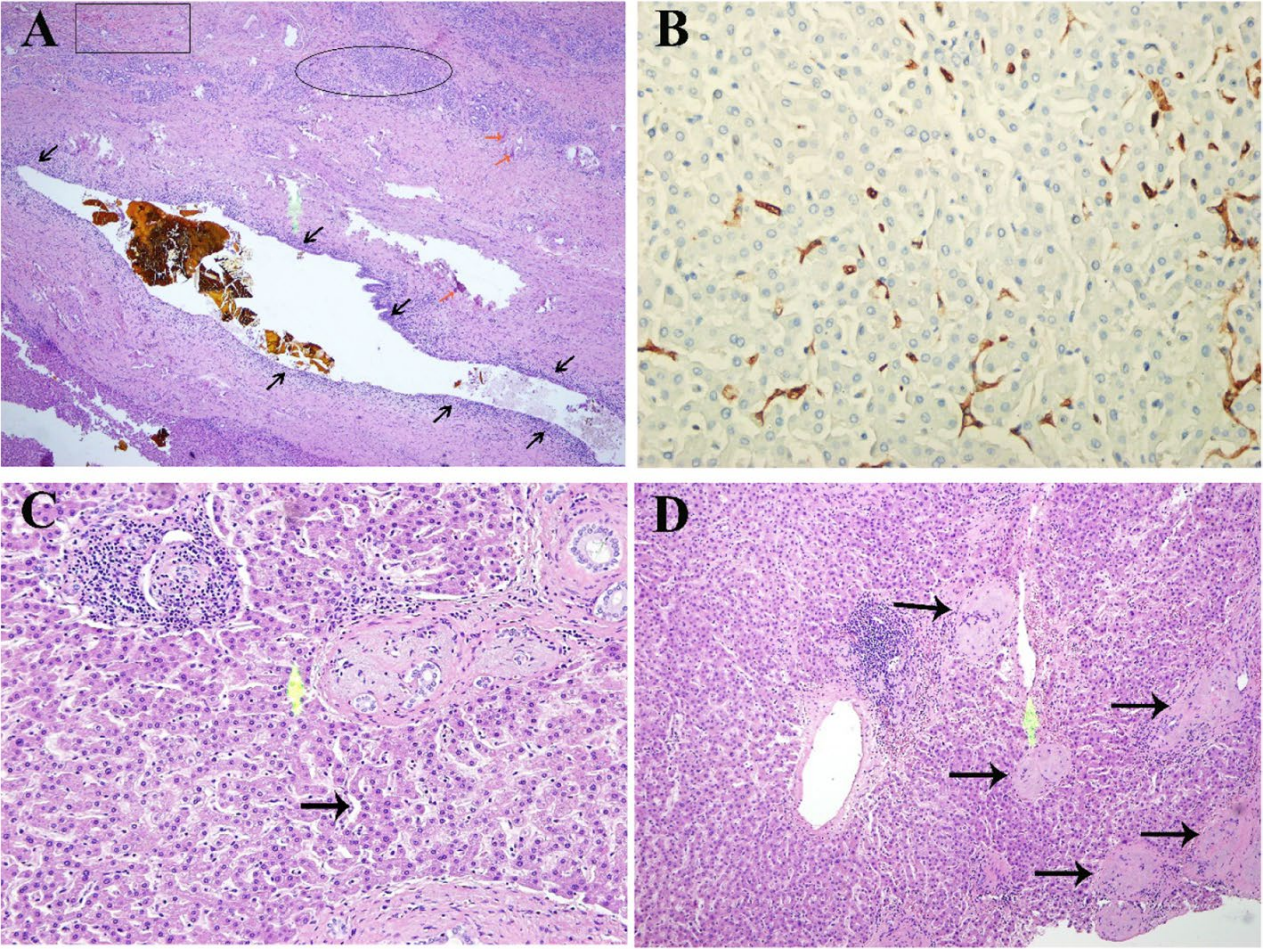
Histological findings of liver biopsy specimens (**A**) Bile duct epithelial hyperplasia, inflammatory cell infiltration (black arrow), necrosis of hepatocytes (orange arrow), formation of the lymphoid follicle (blank irregular round), and local liver fibrosis (blank rectangle). **B** CD34 immunostaining of liver biopsy revealed the presence of positive CD34 in the subendothelial space of central veins and perivenular zones. **C** Local hepatic sinusoid was highly dilated (black arrow), and the hepatic plate atrophy disappeared. **D** Central hepatic vein occlusion and congestion, the branches fibrosis and occlusion, thickened its wall and disordered hepatic plate arrangement (black arrow)

No obvious abnormality was reflected in her laboratory findings during admission (Table 1). Viral serological testing was reported negative for hepatitis A, hepatitis C, and HIV, and the patient was immune to hepatitis B. Serological screening tests for autoimmune diseases were also negative. Atrophy of the left lateral lobe of the liver, hepatolithiasis with dilatation of the bile duct, thickening of the bile duct wall was obtained from abdominal MRI (Fig. 1B). The patient was diagnosed with left hepatolithiasis, left extrahepatic lobe atrophy, and high-risk hypertension. The liver function reserve of the patient was determined to be Child–Pugh class A. The patient voluntarily gave consent and signed a written informed consent form for elective surgery. Under general anesthesia, the patient underwent successful laparoscopic left lateral hepatectomy and bile duct exploration. Histology reported dilated bile duct epithelial hyperplasia, infiltration of inflammatory cell infiltrate, necrosis of hepatocytes, formation of the lymphoid follicle, and local liver fibrosis (Fig. 2A). The patient received smooth postoperative treatment.

**Table 1:** Alteration in laboratory and serology data during treatment

Laboratory exam	Reference Range, Adults^a^	On admission	At POD 2	At POD 4	At POD 8 (After ALSS)	At POD 21 (Date of discharge)
WBC (× 10 9 /L)	3.50–9.50	3.54	9.54	6.61	9.45	2.89
HGB (g/L)	115–150	124	100	107	86	77
PLT (× 10 9 /L)	101–320	178	163	63	9	300
hsCRP (mg/L)	0–5.00	< 0.5	18.23	36.41	15.77	12.66
AST (U/L)	13–35	26	2743	4675	83	63
ALT (U/L)	7–40	18	2843	6307	653	43
TBIL	0.0–23.0	20.4	81.2	93.2	108.8	80.7
DBIL	0.0–8.0	4.9	34.2	46.7	34.6	65.7
IBIL	0.0–20.0	15.5	47	46.5	74.2	15.0
LDH	120–250	212	1982	1606	482	249
ALP (U/L)	50–135	95	118	145	82	149
ALB (g/L)	40.0–55.0	43.4	34.3	33.8	31.3	26.3
GLU (mmol/L)	3.90–6.10	4.71	3.86	6.11	6.7	5.74
PT(sec)	9.8–12.1	10	38.7	31.9	20.9	14.5
PT% (%)	70.0–130.0	129.4	13.7	17.4	30.8	55.7
PT-INR	0.96–1.16	0.91	3.7	3.03	1.95	1.33
D-dimer (mg/L FEU)	0.00–0.55	0.4	35.33	27.01	13.17	4.56

^a^Reference values are affected by many variables, including the patient population and the laboratory methods used. The ranges used at Sichuan Provincial People’s Hospital are for adults

The patient’s liver function significantly deteriorated on the second day after the operation (Table 1), coagulation function test showed PT 38.7 s (9.8–12.1 s), activated partial thromboplastin time 42.8 s (23.3–32.5 s) and D-dimmer 35.33 mg/L, indicating acute liver injury. Coagulation dysfunction developed following operation was managed with administration of 3 units of Fresh-frozen plasma (FFP) to supplement coagulation factors. However, the patient’s clinical condition progressively worsened at POD4, and the patient suddenly became manic, gibberish, and subsequently confused. Physical examination revealed severe icterus of the sclera and skin, disturbances in consciousness in the patient, and Babinski^’^s sign was positive in the right limb. There was continuous regression in the patient^’^s clotting function and liver biochemistry (Table 1); Additional blood tests showed lactate 5.9 mmol/L (0.5–1.7 mmol/L), blood ammonia 328.9umol/L(18-72umol/L), PH 7.452, PO_2_ 104.2mmhg, PCO_2_ 24.2mmhg, HCO_3_^−^ 20.4 mmol/l, base excess -2.9 mmol/l, K^+^ 2.6 mmol/L. Consequently, the patient was admitted to the Intensive care unit (ICU) with the diagnosis of ALF and grade II liver encephalopathy. Symptomatic treatment included prophylactic antimicrobial therapy, FFP, human serum albumin, platelet transfusion, and nutritional liver support. For the management of hyperammonemia and hypokalemia, lactose enemas in combination with intravenous infusion of potassium chloride and a mixture of aspartic acid, ornithine, and arginine were administered.

To identify the etiology of ALF, detailed medical history of the patient was recorded by the patient and her family, whereby they admitted that the patient had taken the GS (Tusanqi) 2–3 g every 24 h for one and a half years. A liver biopsy was conducted for diagnosis. CD34 immunostaining of liver biopsy (200 ×) revealed that positive CD34 was present in the subendothelial space of central veins and perivenular zones (Fig. 2B). Hematoxylin–eosin staining of liver biopsy (200 ×) results showed highly dilated hepatic sinusoid, disappeared hepatic plate atrophy and some portal inflammatory (Fig. 2C). Further, the central venous lumen was irregular and became smaller, the branches of which were fibrosis and occlusion, and its wall was thickened (Fig. 2D). Biopsy results, laboratory findings, and clinical course confirmed the diagnosis of DILI.

**Fig. 3:**
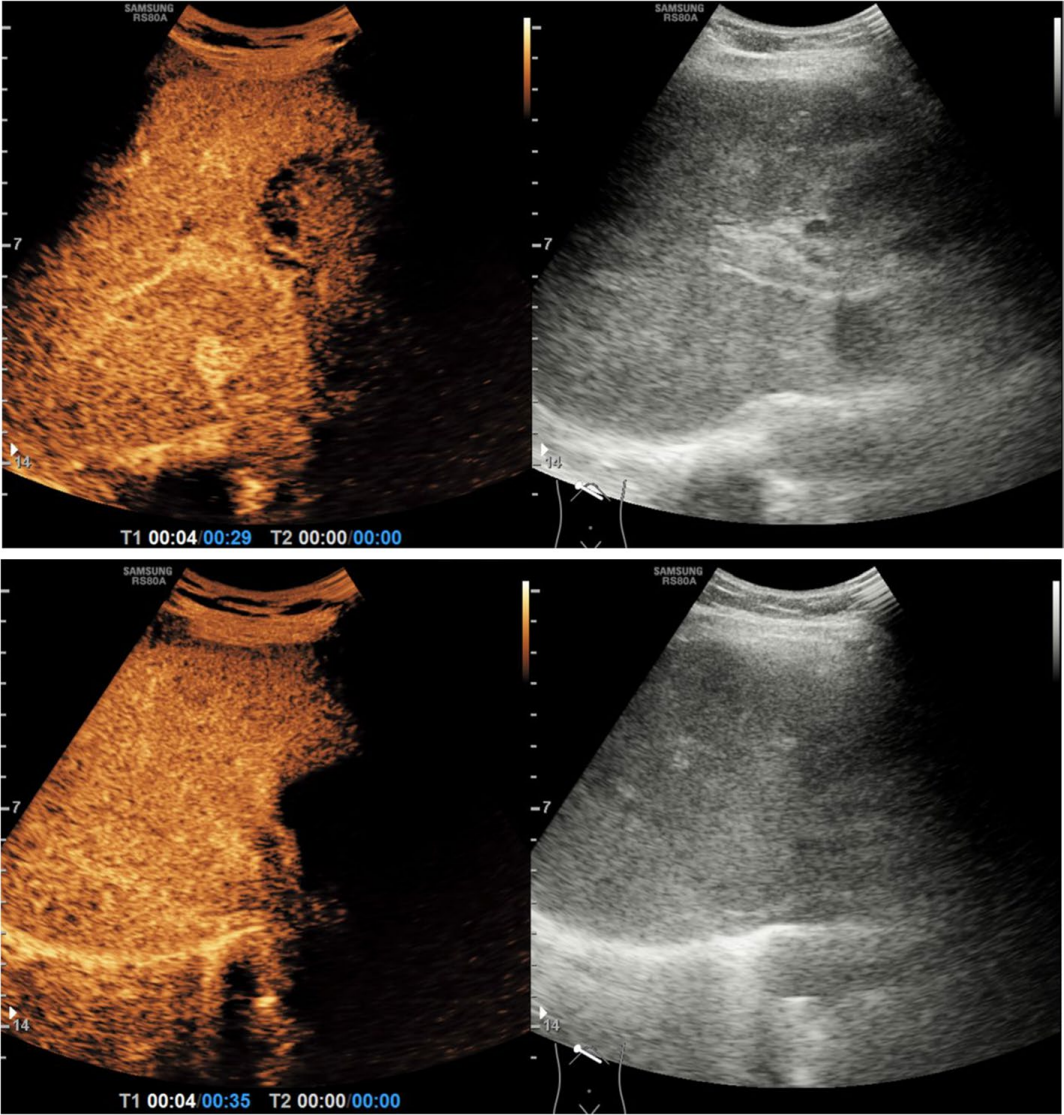
Contrast enhanced ultrasonography after acute liver failure. The echo of liver parenchyma is not uniform, attributed to the high pressure of the hepatic sinusoid. Portal vein perfusion was slightly delayed, there was no hepatic artery-portal vein fistula, and no thrombosis was found in the portal vein

**Fig. 4:**
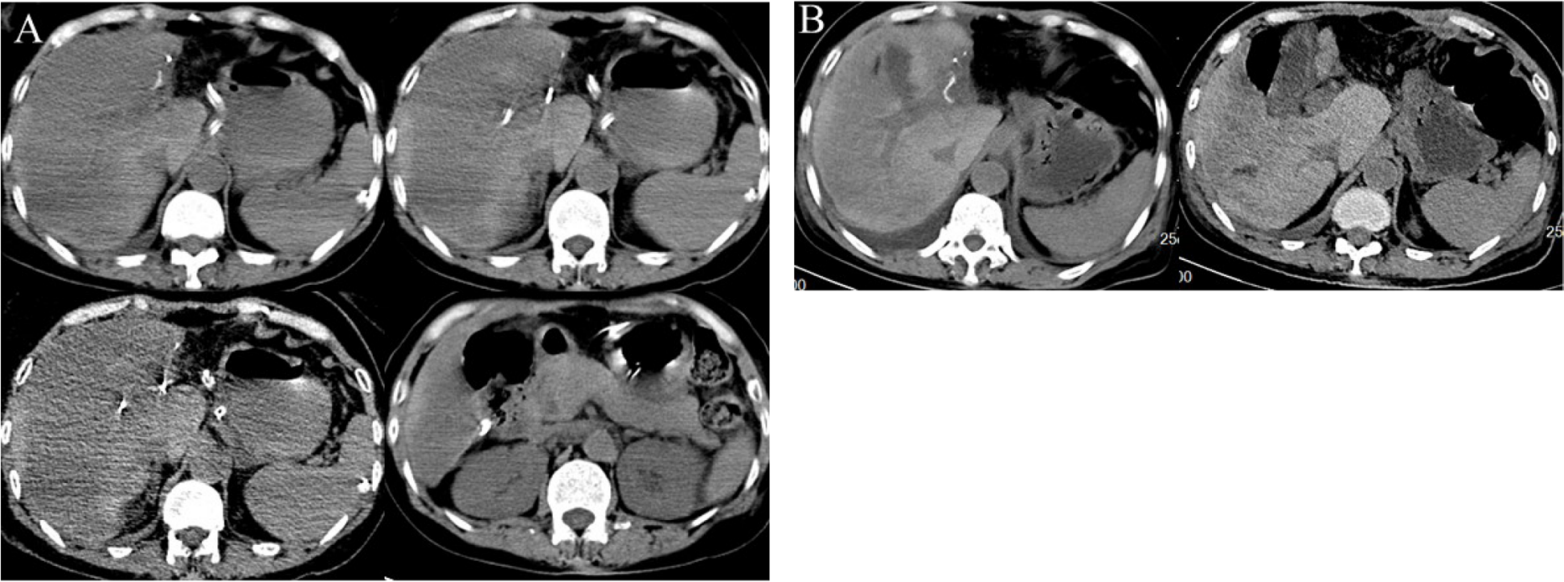
**A** Emergency abdominal CT showing marked liver atrophy and broadly heterogeneous hypoattenuating areas implied heterogeneous hepatic necrosis.** B** Fourteen days after the operation, follow-up CT claimed the disappearance of the heterogeneous hypoattenuating areas coupled with the recovery of the patient’s liver volume

Five days after the operation, an emergency contrast-enhanced ultrasonography confirmed a slight delay in the portal vein perfusion phase, absence of hepatic artery-portal vein fistula, and absence of thrombosis in the portal vein (Fig. 3). Broadly heterogeneous hypo-attenuating areas observed by abdominal CT indicated massive liver necrosis (Fig. 4A). The patient was in a shallow coma. The patient underwent one session of Double plasma molecular absorb system treatment, an ALSS, combined with symptomatic treatment. Following therapy, significant improvement was noted in her clinical signs and symptoms, which was substantiated by the laboratory parameters (Table 1), and follow-up CT also demonstrated that the degree of liver injury was greatly alleviated, the heterogeneous hypoattenuating areas disappeared, and liver volume also recovered (Fig. 4B). Finally, in the third week post operation, the patient got discharged from the hospital and followed up at the outpatient clinic. The patient was in a generally good condition, and no recurrence of DILI and development of liver cirrhosis was reported as of the last follow-up visit at two years after the discharge.

## Discussion and conclusions

DILI is referred to as the liver injury resulting from all types of prescription or non-prescription drugs, including Nonsteroidal Anti-inflammatory Drugs, antimicrobials, Traditional Chinese medicines, natural medicines, health products, and dietary supplements [4–6]. It is often encountered in clinical practice with an incidence of 14–19 cases per 100,000 per year in developed countries, the acute DILI accounts for about 20% of hospitalized patients with acute liver injury [7].

In China, 38 Chinese herbal medicines contain pyrrolizidine alkaloids (PAs), with GS being the most widely used. Two hepatotoxic PAs, namely senecionine and seneciphylline, were indentified in GS by using the HPLC–UV–MS analytical method [1]. These PAs are metabolic activated in the liver through CYP (P450 cytochrome) to electrophilic pyrrolic metabolites. The reactive metabolites generated in situ react with hepatocellular proteins. The resulting protein adduction causes hepatocyte damage by a variety of genotoxicities, including DNA binding, DNA cross-linking, DNA–protein cross-linking, sister chromatid exchange, chromosomal aberrations, mutagenicity, teratogenicity, and carcinogenicity [8]. Furthermore, PAs can decrease glutathione (GSH) in sinusoidal endothelial cells (SEC). This enhanced oxidative stress also can activate hepatic stellate cells [9]. Another study suggested that the main manifestations of PA-induced liver toxicity were progressive damage to SEC, loss of all cell types that comprise the sinusoidal wall, severe central vein (CV) endothelial cells^’^ damage, decreased numbers of Kupffer cells in the lobule, recruitment of monocytes into the lobule and adherent to the CV endothelium, subendothelial and advential fibrosis of CVs, and damage of the CV endothelium with subendothelial hemorrhage [10]. Interestingly, the observed pyrrole–protein adducts in the blood of rats treated with GS appeared to gradually vanish from 100% (day 1) to 64.5% (week 1), 15.2% (week 2), 10.7% (week 3), and undetectable (week 4) after dosing [1]. Thus, only long-term consumption of GS can cause DILI, finally leading to HSOS or liver cirrhosis.

Based on the course of disease, DILI is classified into acute DILI and chronic DILI, the diagnostic criteria of chronic DILI should meet the following conditions: within 6 months after DILI occurs, serum ALT, AST, ALP, or TBIL still remain abnormal, or there is radiographic and histological evidence for portal hypertension or chronic liver injury [11]. The clinical manifestations of acute DILI are usually non-specific. The incubation period varies greatly across individuals, ranging from a few days to several months, and mostly depends on the dose and duration of consumption of the drugs. Most patients with DILI may have no significant symptoms and only manifest the liver biochemical indexes increased in varying degrees such as serum ALT, AST, TBIL, DBIL and ALP [11]. Detection of DILI is more challenging when the patient is suffering from chronic liver diseases. Atypical symptoms and signs of liver injury and portal hypertension may be attributed by patients and clinicians to chronic liver disease, not DILI.

In the absence of any specific clinical characteristics and biological markers of DILI, the diagnostic strategy mainly adopts the DILI algorithm recently proposed by ACG in combination with the Council for International Organizations of Medical Sciences–Roussel Uclaf Causality Assessment Method (RUCAM) [5, 12]. In our case, according to the DILI algorithm recommended by ACG, initial screening with hepatitis serologies, autoimmune serologies, imaging, and liver biopsy were accomplished. Compiling all the evaluations, the viral and autoimmune hepatitis, hepatic ischemia and metabolic disorders were reasonably excluded. Application of the RUCAM to our case estimated the patient’s RUCAM score as 6 (1: Drug start > 90 days, 2: Decrease ≥ 50% in 30 days, 1: Age > 55, 2: Reaction in product label) [5], which indicated a “probable” relationship with GS. These findings collectively helped to confirm a diagnosis of DILI caused by GS.

ALF is a rare and severe consequence of abrupt hepatocyte injury, resulting in a consistent pattern of rapid-onset elevation of aminotransferases, coagulopathy and encephalopathy within a short period of time [13]. Idiosyncratic DILI is the second most common cause of ALF after paracetamol [14]. Only 10% of all patients with DILI develop ALF, with the potential for liver transplantation or death. When combined with chronic liver disease, once DILI occurs there is a higher risk for the appearance of ALF or even death. While the occurrence of ALF in DILI is mainly associated with drug-induced hepatotoxicity and aggravating inducing factors, including surgery [15, 16].

Identification of GS or other factors as the reason for ALF, in this case, is a quite complex issue because abnormal liver enzymes occur after partial hepatectomy; several factors were involved and must be taken into consideration. However, the following aspects presume that ALF in our patient was not caused by underlying diseases and surgery. First, the patient had no history of drinking and was found to be negative for the serological tests for infectious hepatitis, HIV, or other viruses, and had no history of chronic portal hypertension. Autoimmune diseases were also excluded. It is considered that the other liver diseases and hypoxic hepatitis possess less propensity to develop ALF. Meanwhile, intraoperative exploration found that intrahepatic bile duct stones were mainly involved in the left lateral lobe of the liver, the common bile duct and the right liver were not affected by atrophy and obstruction. There was no abnormal liver function before operation. Therefore, it is not considered that chronic left localized hepatolithiasis can affect the function of the right liver and leads to ALF. Moreover, surgery is a big trigger, not a cause. During the operation, the blood flow into the liver was blocked two times for a total of 35 min with the help of the Pringle operation, satisfying the safe occlusion time of 120 min [17]. The possibility of hepatic ischemia resulting from the intraoperative amputation of the hepatic pedicle and the formation of intrahepatic venous thrombosis can be effectively ruled out by conducting postoperative contrast-enhanced ultrasonography (Fig. 3). Only the diseased left external lobe of the patient’s liver was removed, which was well below the safe limits of FLR after liver resection (30% to 50% of the future liver remnant (FLR)/CT-based nontumor liver volume or formula-based liver volume) [18]. After excluding other potential etiologies, GS-induced DILI was the most probable cause of ALF in this case.

For this patient, she was a 63 years old woman. She had been taking GS (2–3 g once per day) for a year and a half, and have primary hepatolithiasis for more than six years. Therefore, she might have higher risk for the appearance of ALF. In the current case, consumption of pyrrolizidine alkaloids-containing GS is prone to develop chronic DILI, manifested as HOSO, which is associated with hepatic megalocytosis, ascites, hyperbilirubinaemia, and hemorrhagic necrosis [19]. The current case is the first report in which GS induces acute DILI, developing the most serious form of DILI, namely ALF. In addition, Her clinical features and prognosis were different from GS related HSOS, which presented jaundice, right upper quadrant pain, portal hypertension, slow progress towards ALF and eventually led to liver cirrhosis [2].

In conclusion, physicians should be aware of the potential liver toxicity of GS. In case of patients with a history of taking GS and other hepatotoxic drugs, strict vigilance should be paid to DILI. Liver biopsy remains the important method for the diagnosis of DILI. Preoperative investigation of the reserve capacity and compensatory capacity of liver function for patients with DILI could avoid postoperative ALF as far as possible. For the treatment of DILI-induced ALF, the drug should be withheld instantly, symptomatic treatment, ALSS and liver transplantation should be actively implemented.

## Data Availability

All data generated or analysed during this study are included in this published article.

## Abbreviations

DILI: Drug-induced liver injury
ALF: Acute liver failure
HSOS: Hepatic sinusoid obstruction syndrome
ALSS: Artificial liver support system
CT: Computed tomography
HIV: Human immunodeficiency virus
MRI: Magnetic Resonance Imaging
POD: Postoperative Day
WBC: White blood cell count
HGB: Hemoglobin
PLT: Platelets
hsCRP: High sensitivity C-reactive protein
AST: Aspartate aminotransferase
ALT: Alanine aminotransferase
TBIL: Total bilirubin
DBIL: Direct bilirubin
IBIL: Indirect bilirubin
LDH: Lactate dehydrogenase
ALP: Alkaline phosphatase
ALB: Albumin
Glu: Glucose
PT: Prothrombin time
